# NLRP3 Inflammasome Plays an Important Role in the Pathogenesis of Collagen-Induced Arthritis

**DOI:** 10.1155/2016/9656270

**Published:** 2016-03-02

**Authors:** Yongfeng Zhang, Yi Zheng, Hongbin Li

**Affiliations:** ^1^Department of Rheumatology and Immunology, Beijing Chao-Yang Hospital, Capital Medical University, No. 8 Gongren Tiyuchang Nanlu, Chaoyang District, Beijing 100020, China; ^2^Department of Rheumatology, The Affiliated Hospital of Inner Mongolia Medical University, Huhhot 010050, China

## Abstract

*Objective*. To investigate the relationship between NLRP3 and the pathogenesis of collagen-induced arthritis.* Methods*. We used the collagen-induced arthritis (CIA) mouse model. The mice were divided into two groups: the model group (CIA, *n* = 16) and the control group (Normal, *n* = 8). The mice were sacrificed seven weeks after immunization. The arthritis score and imaging evaluation (X-rays, Micro-CT, and MRI) were performed. Synovial tissue NLRP3 expression and peripheral blood cytokine levels were analyzed.* Results*. The arthritis score (6.00 ± 2.52), imaging score (4.63 ± 0.92), and synovial tissue NLRP3 expression (4.00 ± 2.03) significantly increased in the CIA mice. The expression of synovial NLRP3 was positively correlated with arthritis clinical and radiographic scores (*r* = 0.792 and *r* = 0.669, resp.).* Conclusions*. The synovial NLRP3 expression increased at the early onset of RA. Synovial NLRP3 expression level was correlated with the clinical arthritis severity and extent of radiological destruction, suggesting that NLRP3 is involved in the pathogenesis of RA.

## 1. Introduction

Rheumatoid arthritis (RA) is a systemic chronic and progressive autoimmune disease. IL-1 families, such as IL-1*β*, IL-18, and IL-33, are important proinflammatory cytokines that are involved in the pathogenesis of RA [1]. The antagonists that target these factors, such as soluble IL-1 receptor, IL-1 receptor antagonist, and IL-6 monoclonal antibodies, are widely used in the treatment of RA. However, some of the patients are not responsive to these biological agents [2]. Thus, the therapeutic methods that are targeting NLRP3 inflammasome, the upstream factors of IL-1 families, may provide a new means for the treatment of RA. Studies show that small molecule compounds MCC950 can reduce the severity of experimental autoimmune encephalomyelitis and multiple sclerosis in mice by blocking NLRP3 activation [3]. NLRP3 is the core protein of “NLRP3 inflammasome” [4–6]. Thus, understanding the relationship between NLRP3 and the pathogenesis of RA is a key point for deeper understanding of the “NLRP3 inflammasome signaling pathways” and the pathogenesis of RA and may provide new targets for the treatment of RA.

Intracellular NLRP3 receptor detects cytoplasmic intracellular pathogens and danger signals. NLRP3 is composed of three domains, LRR (leucine-rich-repeat), NACHT, and PYD (pyrin domain). NACHT is the major structure responsible for NLRP3 activation [7]. NLRP3 expression level is high in bone cells, neutrophils, peripheral blood mononuclear cells, and bone marrow-derived dendritic cells in mouse [8, 9]. Once activated, NLRP3 recruits the adapter ASC (apoptosis-associated speck-like protein containing CARD), effector Caspase-1, and Cardinal to form “NLRP3 inflammasome,” which serves as an activation platform for cytokines. Caspase-1 cleaves proinflammatory cytokines, such as pro-IL-1*β*, pro-IL-18, and pro-IL-33, into their active form, IL-1*β*, IL-18, and IL-33. Under physiological conditions, NLRP3 activates appropriate amount of IL-1*β* and IL-18, which are important proinflammatory cytokines and act as an effective host defense against viruses, bacteria, and fungi invasion [10, 11]. Under pathological conditions, NLRP3 overactivation or mutations will lead to autoinflammatory and autoimmune diseases [12, 13].

Current studies show that NLRP3 inflammasome signaling pathways is involved in the pathogenesis of gout and pseudogout. Moreover, NLRP3 expression has been found in the synovial tissues in the patients with osteoarthritis (OA) [14–16]. These studies indicate that NLRP3 inflammasome pathway is involved in inflammatory joint disease; however, the relationship between the NLRP3 inflammasome and RA pathogenesis is still unclear. Wang et al. show that the NLRP3 mRNA expression in peripheral blood mononuclear cells and granulocytes in RA patients is similar to healthy controls [17].

Collagen-induced arthritis (CIA) mouse model is a widely used experimental arthritis model that has many histopathologic features in common with RA. In this study, we measured the NLRP3 expressions in the serum and synovial tissues at the early onset of CIA in mice to investigate the relationship between NLRP3 and the pathogenesis of CIA. Moreover, this is the first study that explored the relationship between the NLRP3 and severity of arthritis from the imaging perspective.

## 2. Materials and Methods

### 2.1. Experimental Materials

Twenty-four male DBA/1 mice, age of four weeks, weighing ~20 g, were purchased from Beijing Vital River Laboratory Animal Technology Limited. Immunization grade bovine type II collagen (lyophilized), Freund's incomplete adjuvant, and Freund's complete adjuvant were purchased from Chondrex (WA, USA). ELISA kit was purchased from BlueGene (Shanghai, China). NLRP3 monoclonal antibodies were purchased from Abcam (Cambridge, UK). Anti-mouse two-step immunohistochemical detection kit was purchased from Zhongshan Golden Bridge Biotechnology Company (Beijing, China).

### 2.2. CIA Mouse Model

CIA mouse model was created in accordance with the Nature Protocols [18]. In brief, bovine collagen II (10 mg) was dissolved in 0.1 mmol/L acetic acid solution at a final concentration of 3 mg/mL. Equal volumes of dissolved bovine collagen II and complete Freund's adjuvant emulsified on ice to a final concentration of 1.5 mg/mL. During the first immunization (day 0), 100 *μ*L collagen was delivered by intradermal injection at the base of the tail. Booster immunization (the third week) was induced by intradermal injection at the base of the tail with 50 *μ*L collagen. Arthritis commonly developed at 3-4 weeks after immunization and peaked at 7 weeks. According to the results of preliminary experiments, the mice were divided into two groups, the model group (CIA, *n* = 16) and control group (Normal, *n* = 8). All mice were sacrificed at week 7 after immunization. The study was approved by the ethics committee.

### 2.3. Clinical Evaluation

The diet, hair color changes, and general activity of each mouse were monitored daily after the booster immunization. The body weight of the mice was recorded every week. The joint swelling of CIA mice was observed daily, and the clinical score and the number of swollen joints were evaluated. Arthritis scoring criteria were as follows: 0 points, no joint swelling; 1 point, 1-2 toes swelling; 2 points, ≥3 swollen toes; 1 point each for palm or wrist or ankle swelling, respectively. The highest score for each paw was 4 points and the total score for each mouse was 16 points. Joint count method was as follows: each toe was counted as 1 joint; palm/ankle/wrist was counted as 1 joint. Each paw had a total of seven joints, and each mouse had a total of 28 joints.

### 2.4. Imaging Evaluation

At the end of the experiment, 8 CIA mice and 4 Normal mice were randomly selected for radiography of both hind limbs. The anteroposterior plain radiograph was acquired with Siemense inveon CT (80 KV, 500 uA). Micro-CT (Siemense inveon CT) coronal plane images were acquired at resolution of 30 *μ*m, FOV of 54.6 mm × 27.4 mm, thickness of 29.86 *μ*m, layer spacing of 29.86 *μ*m, and scan time of 28 minutes and 18 seconds. The images were processed using Inveon Acquisition Workplace software. Radiology grading criteria were as follows: 0, no bone damage; 1, tissue swelling; 2, joint erosion; 3, bone erosion and osteophyte formation [19]. Each mouse received imaging of both hind limbs. The radiographic total score of both hind limbs was the total score for each mouse.

The 8 CIA and 4 Normal mice received MRI (magnetic resonance imaging) radiography (BRUKER, Germany) of both hind limbs to detect the synovial and bone marrow edema. The images were processed using pharmascan 7T imaging systems and Paravision Version software with 20 mm coil. Scanning sequence was T1+enhanced fat-suppressed sequences, with TR of 269.9 ms, TE of 8 ms, FOV of 25 mm, thickness of 0.25 mm, layer spacing of 0.25 mm, and scan time of 5 minutes and 45 seconds. 100 *μ*L of Gd-DTPA (contrast agent) was injected via the tail vein. The bone marrow edema on the MRI image was defined as abnormal high signals in marrow tissues; synovial hypertrophy was defined as abnormal defused high signals in joint space. Mice were injected with 10% chloral hydrate before radiography [20].

### 2.5. Histological Evaluation

At the end of the experiment, four paws of each mouse were fixed in 10% formalin, decalcified with EDTA for six weeks, embedded in paraffin, sectioned (4 *μ*m of thickness), and stained with HE staining. Pathological assessment was performed in accordance with RA synovitis score. Synovial pathological score consisted of synovial lining layer hyperplasia, the degree of inflammation of the lower lining layer, and angiogenesis. Lining layer hyperplasia score criteria were as follows: 0 points, less than 3 layers; 1 point, 3-4 layers; 2 points, 5–7 layers; 3 points, 7 or more layers. Inflammation score criteria were as follows: 0 points, no inflammatory cells; 1 point, scattered inflammatory cells; 2 points, diffuse distribution; 3 points, forming germinal centers of lymphoid follicles. Angiogenesis rating criteria were as follows: 0 points, no angiogenesis; 1 point, mild angiogenesis; 2 points, moderate angiogenesis; 3 points, severe angiogenesis. The total pathological score of each paw was the cumulative scores of the three score systems. The total pathological score of each mouse was the sum of the pathological scores of 4 paws [21, 22].

### 2.6. Evaluation of NLRP3 Expression in Synovial Tissues

Joint tissues were decalcified with EDTA for six weeks, paraffin-embedded, sectioned at 4 *μ*m of thickness, and stained with two-step immunohistochemical staining kit. NLRP3 mainly expressed in the cytoplasm and the positive staining was defined as the presence of brown granules in cytoplasm. The hyperplasia of the synovial lining and the lower lining layer in each slice was evaluated at 10 × 40 magnification. The semiquantitative assessment of the ratio of positive cells and staining intensity was performed in accordance with the literature [23]. The ratio of positive cells was graded as follows: 0 points, no staining or a single positive staining cell; 1 point, the positive rate of <10%; 2 points, the positive rate of 11% to 50%; 3 points, the positive rate of 51% to 80%; 4 points, the positive rate of >80%. The staining intensity was graded as follows: 0 points, consistent with the background color; 1 point, pale yellow, slightly higher than the background color; 2 points, brown, significantly higher than the background color; 3 points: tan. The total score was the sum of the scores of the positive cell ratio and the staining intensity. Based on the total scores, the NLRP3 expression was divided into four levels: negative, ≤10% positive cells, regardless of staining intensity; weak expression, 3 points; moderate expression, 4-5 points; strong expression, 6-7 points. Five fields were randomly selected in each slice and the mean value of the five fields was defined as the total score of each slice.

### 2.7. Evaluation of Peripheral Blood Cytokine Levels

At end of the experiment, the mice were sacrificed and orbital blood was collected. NLRP3, IL-1*β*, IL-18, and IL-33 levels in peripheral blood were detected using ELISA kit and analyzed.

### 2.8. Statistical Analysis

Data analysis was performed using SPSS11.5 statistical software. Body weight between groups was analyzed using Student's* t*-test (normally distributed data). Cytokines between groups were compared using Mann-Whitney* U* test and expressed as median. *P* < 0.05 was considered statistically significant. NLRP3 serum levels and arthritis clinical scores were analyzed using Pearson's correlation analysis. Joint clinical score, radiographic score, and joint NLRP3 expression were analyzed suing Pearson's correlation analysis.

## 3. Results

### 3.1. CIA Clinical Evaluation

There was no mortality in CIA and Normal mice during the experiment. 16 mice in CIA group developed CIA. The mice in Normal group showed increased weight gain, active, shiny hair, and easy movement. The mice in CIA group showed various degrees of redness and swelling in the hind limbs, dull hair and hair loss, fatigue, weariness, and slower or even declined weight gain at 3-4 weeks after immunization.

**Figure 1:**
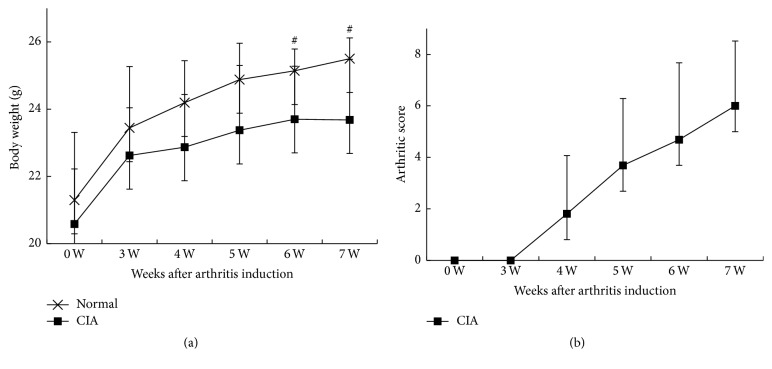
Clinical evaluations. (a) The changes in the body weight in the CIA group and the Normal group. The body weight in CIA group significantly reduced at 6-7 weeks after immunization, compared with the Normal group (*P* = 0.005 and *P* = 0.002, resp.). (b) The arthritis clinical scores in CIA group significantly increased at 4 weeks after immunization and peaked at 7 weeks after immunization. ^#^ *P* < 0.05, compared with Normal group.

Some of the CIA mice had inflammatory ulcers at the injection site at 3 weeks after immunization, and the ulcers formed hard crusts at 4 weeks after immunization. The mice in the Normal groups had steady weight gain during the experiments, whereas the weight gain slowed down in the CIA mice 3 weeks after immunization. The CIA mice showed weight loss at 6 weeks after immunization. The body weights of CIA mice at 6 and 7 weeks after immunization were 23.70 ± 1.57 g and 23.68 ± 1.80 g, respectively, which were significantly lower than Normal group 25.14 ± 0.65 g (*P* = 0.005) and 25.50 ± 0.62 g (*P* = 0.002), respectively (Figure 1(a)).

CIA mice had toe swelling and limited mobility 3-4 weeks after immunization. The arthritis scores reached a peak at 7 weeks after immunization (6.00 ± 2.52). The maximum number of affected joints was 15 and joint deformity occurred in some CIA mice. Normal controls did not show joint swelling (Figure 1(b)).

### 3.2. Imaging Evaluations

MRI scans were performed at the end of the experiment. MRI with T1+enhanced fat-suppression sequences showed that the tarsal, metatarsal, distal tibia, and surrounding soft tissues were clear and complete in Normal mice, with no soft tissue/bone marrow edema and synovial thickening. The articular soft tissues in the CIA group showed a large area of abnormal high signals, suggesting soft tissue edema; in addition, abnormally high signals were seen at the articular surface and joint space, suggesting bone marrow edema and synovial thickening.

**Figure 2:**
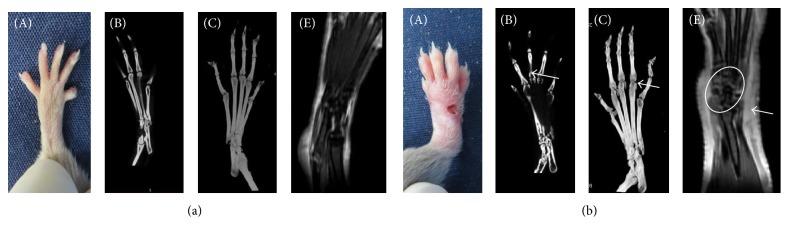
The imaging evaluations. (a) Images of the Normal mice. (A) Clinical imaging; (B) Micro-CT coronal imaging; (C) three-dimensional imaging; (E) MRI imaging. (b) Images of the CIA mice. (A) The clinical imaging showed four toes, palm and ankle swelling, and palm skin ulceration; (B) CIA mouse Micro-CT coronal imaging revealed cystic destruction at metacarpophalangeal joints; (C) three-dimensional Micro-CT imaging revealed bone thinning at the metacarpophalangeal and proximal interphalangeal joints; (E) there was significant signal enhancements at a large area of soft tissues surrounding the joints, as indicated by the arrows. In addition, there was visible signal enhancement at articular surface of the annular region, suggestive of synovitis and bone marrow edema.

X-rays revealed no soft tissue swelling, complete bone structure at articular surface, and clear toe joint space in Normal mice, whereas the CIA mice showed soft tissue swelling, bone erosion, and osteophyte formation at joint surface. The three-dimensional Micro-CT imaging showed bone thinning, cystic resorption, and bone erosion at the metatarsophalangeal joint surface in the CIA mice. The joint imaging score in CIA mice at the end of the experiment was 4.63 ± 0.92 (Figures 2 and 4).

### 3.3. Histopathological Evaluation

**Figure 3:**
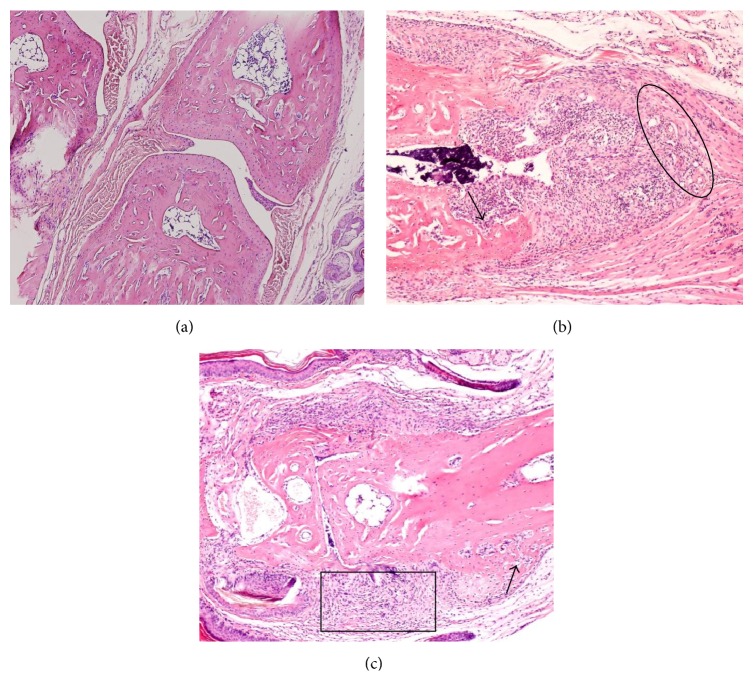
Histopathological evaluation. (a) The Normal mice showed normal joint space at the interphalangeal joint, 1–3 synovial cell layers, and smooth articular cartilage surface. (b), (c) CIA mice showed diminished interphalangeal joint space and diffuse infiltration of inflammatory cells (square area); significant proliferation of synovial cells and synovial cells invasion into cartilage and bone (arrow); visible neovascularization at synovial hyperplasia areas and inflammatory cell infiltration at perivascular areas (ring area). Magnification (×200).

Normal group showed no inflammatory cell infiltration at the joint tissues, 1-2 layers of synovial cells, smooth cartilage surface, and intact articular structures. CIA group showed 10 or more layers of synovial cells, proliferated synovial tissues extended into the joint cavity, and pannus formed in the articular cartilage and bone with an average score of 3.63 ± 1.50. Meanwhile, CIA mice showed diffused infiltration of inflammatory cells at the joint tissues and the formation of lymphoid follicles, with an inflammatory cell infiltration score of 3.00 ± 1.03. In addition, CIA mice showed visible neovascularization within the synovial tissues and inflammatory cells infiltration at the perivascular areas, with an angiogenesis score of 1.43 ± 1.36 (Figure 3).

### 3.4. Synovial Tissue NLRP3 Expression and Correlation Analysis

Normal group showed no NLRP3 protein expression. CIA group showed enhanced NLRP3 expression, mainly expressed in the cytoplasm of the synovial cells. NLRP3 average score in CIA group was 4.00 ± 2.03.

**Figure 4:**
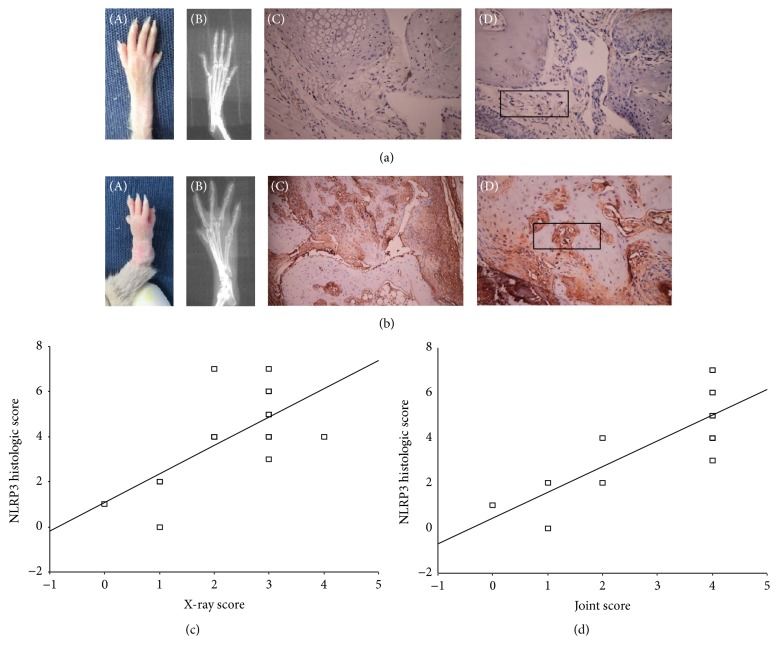
Synovial tissue NLRP3 expression and correlation analysis. (a) Normal mice. (A) No soft tissue swelling; (B) X-rays imaging showed no bone destruction and no joint space narrowing; (C, D) no significant synovial hyperplasia. (b) CIA mice. (A) Paw imaging showed significant articular soft tissue swelling; (B) X-rays showed the bone destruction and joint space narrowing at the proximal interphalangeal and metacarpophalangeal joint surface and visible metacarpophalangeal joint dislocation; (C) NLRP3 immunohistochemical staining showed significant NLRP3 expression at synovial proliferation areas and subchondral vasculitis areas; (D) square area showed increased NLRP3 expression in the synovial vascular endothelial cells that invaded the cartilage. (c) NLRP3 histology score and joint imaging score were positively correlated. (d) NLRP3 histology score and arthritis clinical score were positively correlated. Magnification (×200).

Radiography was performed in two hind limbs of 8 CIA mice (total of 16 paws). The correlation of the clinical score/radiographic score and NLRP3 immunohistochemistry of each paw was analyzed. NLRP3 immunohistochemistry score and clinical score were positively correlated (*r* value of 0.792, *P* = 0.000); NLRP3 immunohistochemistry score and imaging score were positively correlated (*r* value of 0.669, *P* = 0.005) (Figure 4).

### 3.5. Peripheral Blood Cytokine Levels and Correlation Analysis

**Figure 5:**
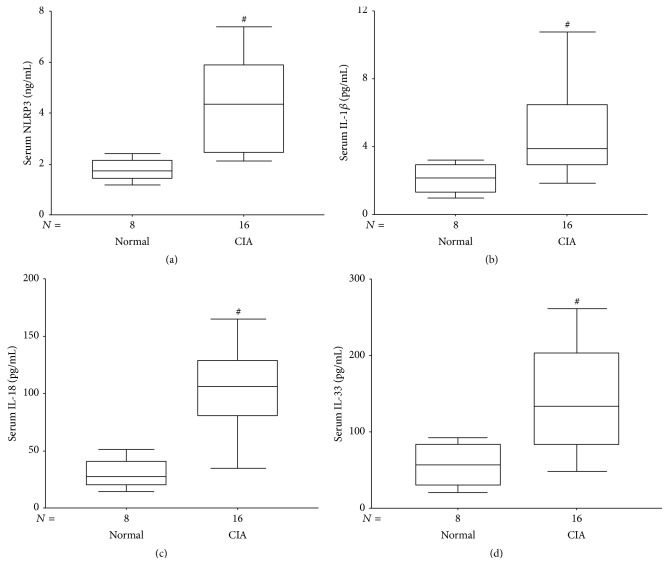
Serum cytokine levels in Normal and CIA groups. (a) The median serum NLRP3 levels in the Normal and the CIA groups were 1.73 ng/mL and 3.44 ng/mL, respectively, *P* = 0.001. (b) The median serum levels of IL-1*β* in the Normal and the CIA group were 2.14 pg/mL and 2.82 pg/mL, respectively, *P* = 0.005. (c) The median serum levels of IL-18 in the Normal and the CIA group were 27.67 pg/mL and 30.60 pg/mL, respectively, *P* = 0.000. (d) The median serum levels of IL-33 in the Normal and the CIA group were 61.09 pg/mL and 67.15 pg/mL, respectively, *P* = 0.004. ^#^ *P* < 0.05, compared with the Normal group.

The serum NLRP3, IL-1*β*, IL-18, and IL-33 levels in CIA were significantly higher than the Normal group (*P* = 0.001, *P* = 0.005, *P* = 0.000, and *P* = 0.004, resp.) (Figure 5).

NLRP3 serum level and clinical and radiological scores in CIA mouse did not show correlation (*r* = 0.165 and *r* = 0.237, resp.).

## 4. Discussion

NLRP3 inflammasome activates caspase-1, which in turn cleaves pro-IL-1*β*, pro-IL-18, and pro-IL-33 to generate the active forms of proinflammatory cytokines that are involved in inflammation [10–12, 24]. Our previous study indicated that caspase-1 inhibitor treatment reduced serum IL-1*β*, pro-IL-18, and pro-IL-33 levels in CIA mice. Studies on gout pathogenesis have demonstrated that a single crystal of sodium urate can change NLRP3 configuration, resulting in NLRP3 activation and release of a large number of proinflammatory cytokines, including IL-1*β*, which participate in the pathogenesis of arthritis [25]. However, there are controversial research reports on NLRP3 and RA. This study shows that NLRP3 expression at synovial membranes in the CIA mice significantly increased and is associated with the severity of arthritis, suggesting the involvement of NLRP3 in the pathogenesis of arthritis.

In this study, we used a mouse model of CIA to investigate the relationship of NLRP3 and arthritis. Mice underwent MRI scans at 4 weeks after the booster immunization and the CIA group showed the imaging features of early onset of arthritis, such as articular soft tissue swelling, synovial hypertrophy, and bone marrow edema. HE staining showed significant proliferation of synovial cells and pannus invasion of articular cartilage and bone, visible neovascularization, and perivascular infiltration of inflammatory cells in the synovial tissue. Imaging and pathology revealed that the endpoint of this search was the early onset of CIA. We found that there was no NLRP3 protein expression in Normal group, whereas synovial NLRP3 expression significantly increased in CIA mice. NLRP3 was mainly expressed in the synovial cell cytoplasm, but also in vascular endothelial cells in CIA mice, suggesting the involvement of NLRP3 in synovial cell proliferation and angiogenesis. CIA group NLRP3 serum levels were significantly increased compared with Normal group (*P* = 0.001). The increased synovial and serum NLRP3 expressions strongly suggest that the NLRP3 is involved in pathogenesis of arthritis.

Our study suggests that NLRP3 is involved in arthritis pathogenesis. However, there is no report on whether NLRP3 is related to the severity of arthritis. Our study is the first one that analyzed the correlation of serum and joint NLRP3 expressions and arthritis clinical and radiographic scores. Correlation analysis showed that synovial NLRP3 expression is positively correlated with arthritis clinical and radiographic scores, but serum NLRP3 levels did not show correlation with clinical radiographic scores. Correlation analysis showed that the synovial NLRP3 expression might be directly related to the pathogenesis and the disease severity of arthritis. However, serum NLRP3 expression may not be directly relevant to the arthritis. However, serum NLRP3 expression was significantly higher in CIA mice than that in the Normal group, suggesting that serum NLRP3 may be associated with systemic inflammation.

Rosengren and other studies have shown that NLRP3 is expressed in the synovial membranes in RA and OA, but the expression in RA is significantly increased and NLRP3 is mainly expressed in synovial lining cells [26]. Kolly et al. have found that NLRP3 is mainly expressed in synovial medullary cells, endothelial cells, and B cells, but synovial fibroblasts do not express NLRP3 protein and RNA in RA [27]. Yang et al. show that NLRP3 coding gene rs4353135 mutation is related to the susceptibility to juvenile idiopathic arthritis, inflammatory markers, and response to TNF treatment in Taiwanese [13]. Choulaki et al. show that patients with active RA have increased expression of NLRP3 and NLRP3-mediated IL-1*β* secretion in whole blood cells upon stimulation via TLR3 and TLR4 [28]. Mathews et al. show that genetic variants within the NLRP3 inflammasome complex are related to the susceptibility of RA and the response to anti-TNF treatment in Caucasian patients [29]. However, other animal models and some human studies also show that NLRP3 may be irrelevant to arthritis. Ding et al. show that curculigoside exhibits protective effects on adjuvant-induced arthritis via inhibiting NLRP3 activation in rats [30]. In an antigen-induced arthritis mouse model, NLRP3 inflammasome defect does not affect the incidence of arthritis. CIA mouse model studies also show that there is no difference in the incidence and severity of arthritis between the NLRP3^−/−^ and caspase-1^−/−^ mice and the wild-type mice [31]. Ben Hamad et al. show that NLRP3 (p.Q705K) gene has no effect on RA susceptibility in the French and Tunisian population [32]. Our study shows that NLRP3 is associated with the systemic inflammation in RA and NLRP3 is related to the clinical and radiographic scores.

IL-1*β*, IL-18, and IL-33 are the major NLRP3 downstream effectors and are directly involved in the pathogenesis and progression of RA. Our study shows that serum IL-1*β*, IL-18, and IL-33 in CIA mice significantly increased compared with Normal mice, which are consistent with previous findings. Interleukin antagonists have been used in the clinical practice, but their pharmaceutical characteristics limit their clinical application. Thus, blocking the upstream factors in the proinflammatory signaling pathways will be the new therapeutic targets. Our previous study indicated that caspase-1 inhibitor VX765 prophylactic treatment significantly reduced serum cytokine (IL-1*β*, IL-18, and IL-33) levels and ameliorated the severity and progression of CIA. This study suggests that NLRP3, the key upstream protein in the proinflammatory cytokine-signaling pathway, is associated with the pathogenesis of CIA.

## 5. Conclusions

In conclusion, our study shows that NLRP3 inflammasome signaling pathways is associated with the pathogenesis of CIA. NLRP3 expression increased in the early onset of CIA. This is the first study that shows that the extent of synovial NLRP3 expression is correlated with the clinical severity of arthritis and radiological scores. These findings provide ideas for the treatment of RA.

This study shows the relationship between NLRP3 and arthritis; however, the animal sample size is small and experimental and analytical methods have limitations. In our future study, we will increase the sample size and apply more experimental/analytical method to further study the relationship between the NLRP3 expression in synovial tissue and arthritis pathogenesis.
